# Modelling job support, job fit, job role and job satisfaction for school of nursing sessional academic staff

**DOI:** 10.1186/s12912-018-0290-2

**Published:** 2018-05-24

**Authors:** Leanne S. Cowin, Robyn Moroney

**Affiliations:** School of Nursing & Midwifery, Western Sydney University, Locked Bag 1797, Penrith Soutah DC, NSW 2751 Australia

**Keywords:** Sessional staff, Job satisfaction, Organisational fit, Job support, Job role, Nursing education

## Abstract

**Background:**

Sessional academic staff are an important part of nursing education. Increases in casualisation of the academic workforce continue and satisfaction with the job role is an important bench mark for quality curricula delivery and influences recruitment and retention. This study examined relations between four job constructs - organisation fit, organisation support, staff role and job satisfaction for Sessional Academic Staff at a School of Nursing by creating two path analysis models.

**Methods:**

A cross-sectional correlational survey design was utilised. Participants who were currently working as sessional or casual teaching staff members were invited to complete an online anonymous survey. The data represents a convenience sample of Sessional Academic Staff in 2016 at a large school of Nursing and Midwifery in Australia. After psychometric evaluation of each of the job construct measures in this study we utilised Structural Equation Modelling to better understand the relations of the variables.

**Results:**

The measures used in this study were found to be both valid and reliable for this sample. Job support and job fit are positively linked to job satisfaction. Although the hypothesised model did not meet model fit standards, a new ‘nested’ model made substantive sense.

**Conclusion:**

This small study explored a new scale for measuring academic job role, and demonstrated how it promotes the constructs of job fit and job supports. All four job constructs are important in providing job satisfaction – an outcome that in turn supports staffing stability, retention, and motivation.

**Electronic supplementary material:**

The online version of this article (10.1186/s12912-018-0290-2) contains supplementary material, which is available to authorized users.

## Background

The importance of Sessional Academic Staff in teaching and learning at universities throughout Australia continues to increase [1]. The term Sessional Staff includes casual, associate, adjunct or part-time, ancillary or auxiliary academic staff aiming to capture the transient or temporary state of employment. There are now more sessional staff employed in Australian Universities than full time academics [2]. Attracting and retaining dedicated teaching staff is now crucial to the functioning of some Schools of Nursing in Australia and the provision of a quality curriculum [3]. Nurses of the future are dependent on sessional as well as full time academic staff.

The quality of teaching at tertiary level has never been more important to student outcomes and workforce contributions [4]. High quality teaching, according to Queensland University of Technology [5], should capture students into a learning partnership whereby personal and professional development is inspired, fostered, and ultimately practised as new graduates. Such teaching provides rigorous feedback and evaluation within the learning environment with the support of sessional academic teaching staff being critical to the success of the graduate, particularly in health care professions [6]. However, despite higher numbers of Sessional Staff (SS), less support is available now and it is provided by fewer staff members [6].

Recent liberal directions of universities throughout Australia have involved policies such as ‘uncapped enrolment’ whereby University programs such as the Bachelor of Nursing are able to recruit as many students as they wish [7]. Previously, student numbers were capped and according to the ‘Group of Eight’ [8], universities are calling for a recapping of placements within courses including nursing due to spiralling costs and student quality issues. The ‘Group of Eight’ consists of Australia’s eight leading research Universities [8]. Ever increasing numbers of students have not been matched by staffing and resources [7] leading to greater stress than ever before on academic staff [9]. As stated by Harvey, ‘the main failure of university expansion is the unwillingness to fund it’ [10].

The need for further SS continues to grow as the numbers of full time nursing academics dwindle. Shortages of full time academic staff persists in countries such as the USA and Canada [11], and causes identified for the growing shortages include distressingly high workloads, aging academics, perceived lack of teaching support and inflexible work life [12, 13]. In 2003, the Australian Universities Teaching Committee found that the tertiary education sector had managed the ‘casualisation’ of the teaching workforce ‘quite poorly’ in terms of training and support ([14]p. i). More than a decade on, it remains unclear whether the SS member receives the support, training, resources, and satisfaction they need to continue working in a part time capacity [2]. Over the past 20 years, the numbers of SS have increased to more than the number of tenured staff [2, 14]. SS are employed as lecturers, tutors, and lab demonstrators on a casual or sessional basis. While SS bring ‘flexibility, diversity and financial savings’ ([14] p. i), job satisfaction, support and training remain problematic and retention becomes a critical employment issue. Sessional staff (SS) are people employed on a part time basis for a short period to deliver and assess curricula to university students [13]. McCormack [15], describe this group as teachers employed on a casual, contractual or sessional basis.

Within nursing education, a tension exists between clinical currency and teaching expertise [16]. Do nursing academics, who divide their time between the clinical field and the university environment, provide superior and more relevant nursing education, or do they have more teaching difficulties because of this dual focus? Currently, there are signs of a rise in teaching expertise and a decrease in dual roles of teaching and clinical work as the need for SS increases. This is in contrast to previously held notions of the SS member being unqualified in adult education [17], and being commonly engaged in clinical practice [18]. Evidence from recent workforce assessment reveals some Australian universities are extensively ‘casualised’ and many tasks attributable to the academic role such as journal reviewing, editing, student feedback, and committee attendance are not possible in the timeframe available [19].

Enjoyment of work is an important construct in any workplace but it is probably most important for those people who work casually. If SS are not satisfied with their work, the option of employment elsewhere is potentially much easier than for the full time permanent academic [2]. Indeed, much of the attraction of sessional work centres on a sense of flexibility with work choices [20]. For the organisation to attract the best academics job satisfaction is vital and that assessing and making adjustments to increase job satisfaction is critical [21].

Research into job satisfaction continues to be popular in all organisational studies primarily because of the strong empirical evidence supporting causal relationships between satisfaction with work and retention [22, 23]. Coates et al. [24] found that Australian academics have among the lowest levels of satisfaction in the world. In addition, the connection between satisfaction and performance quality and effectiveness is also important and has significant financial and productivity implications [23]. The workplace of SS varies substantially from that of the fulltime academic in management and work setting. For example, access to office space may be limited or even non-existent. Access to the teaching team may also be varied. Accessing other SS may be easier than accessing fulltime academics. The role of the SS is fraught with issues relating to work flexibility, multiple campus sites, financial reimbursement, and team communication [15].

### Aims

The purpose of this study was to create and test a path analysis model containing the variables of job satisfaction, organisational support, organisational fit, and sessional staff role. In a previous quality improvement project from 2014, the SS raised the subjects of job satisfaction, job fit, job support, and job role as important themes. These four topics were reviewed in the research literature, specifically those with valid and reliable tools. Where possible the shortest tools were sought due to potential sample size limitations and survey length issues. Theoretical support for this model is gained from the well supported notion that increased job satisfaction promotes retention and intrinsic rewards [25, 26], (see extensive literature based on these ‘work and motivation’ theorists). It is hypothesised that: Organisational support, organisational fit, and sessional staff role will positively and significantly contribute to job satisfaction. In this model, job satisfaction is treated as the dependent variable.

## Methods

### Sample

This study was conducted in the School of Nursing and Midwifery at a large multi-campus university in Australia. In 2015–6 approximately 85 fulltime teaching academics and 150 regular SS taught or marked assignments. All SS were emailed an invitation to participate in this study and the invitation was displayed on the SS electronic resource site. The email and advertisement directed potential participants to the survey site www.Qualtrics.com at the end of the second semester for the year – November-December 2015 and again in early 2016. Sixty-six sessional staff attempted the survey in second semester 2015 and 67 in first semester 2016 thereby providing 133 completed surveys for cross sectional analysis. As different subjects are conducted in the semesters, different SS were accessed in each semester thereby allowing us to treat participants as one homogenous group. The online survey precluded missing data, contained a brief outline of the study, a series of short answer questions, and the four scales utilised in this study. The qualitative data gathered will be reported elsewhere.

### Measures

*Global Job Satisfaction* is measured using a six item tool by Pond and Geyer [27] (see Additional file 1). The items assess affective ‘facet free’ responses, and although Quinn and Sheppard originally posited a 5 item scale in 1974 (α = 0.88, M = 3.75), it is the 6 item version by Pond and Geyer in1991 [27] that has been utilised in this study. Psychometrics from the 1991 Pond and Geyer study indicate a mean score of 2.76 (SD 0.92) and an alpha of 0.89 in a sample of 70 non-unionised textile workers. In a more recent study by Gutierrez, Candela and Carver [28] the researchers, using the Pond and Geyer version, reported a mean score for global job satisfaction as 4.18 (SD 0.65) and an alpha of 0.93 in a sample of 570 nursing academics in the USA.

Support for SS is measured using the *Perceived Organisational Support Scale* (POSS) by Eisenberger et al. [29]. Originally, this measure contained 36 items, and was designed to explore employees’ beliefs of how much the organisation they worked for valued their work and their well-being. In 2012 Gutierrez et al. [28] also utilised nine relevant items from the Eisenberger et al. [29] scale for their sample of 570 nursing academics and demonstrated a mean score of 5.20 (SD1.16) by utilising a 7-point Likert scale (1 = strongly disagree, 7 = strongly agree) however, no alpha score is reported. Tourangeau et al. [11]also utilised the POSS nine item 7-point scale for their study of 650 nursing academics, reporting a mean score of 4.09 (SD 1.38). The authors [11] reported an alpha score of 0.93.

For the current study, it was agreed by the research team that only six items of Guitierrez et al., [28]and Tourangeau et al., [11] nine item POSS related to SS. The use of the original phrase – ‘The Organisation’, would be altered to - The School of Nursing & Midwifery (*see* Table 1).

**Table 1:** Perceived Organisational Support Scale (POSS) by Eisenberger et al. (1986)

Item number	Item wording
27	The School of Nursing & Midwifery takes pride in my accomplishments at work.
9	The School of Nursing & Midwifery really cares about my well-being.
1	The School of Nursing & Midwifery values my contributions to its well-being.
4	The School of Nursing & Midwifery strongly considers my goals and values.
23^a^	The School of Nursing & Midwifery shows little concern for me.
20	The School of Nursing & Midwifery is willing to help me if I need a special favour.

^a^- reverse scored

Organisational suitability was measured using the 3 item ‘*Perceived-Person Organization Fit’* scale (PPOF) by Cable and Judge [30]. The internal consistency estimate for the 3-item scale was 0.68 based on a study of 320 job seekers utilising 3 points in time (Time 1 *N* = 320, Time 2 *N* = 96, and Time 3 *N* = 68). In the Gutierrez et al. 2012 study of 1453 nursing academics, a sample of 570 demonstrated a mean score of 3.96 (SD 0.60) and an alpha score of 0.90.

The *Sessional Staff Role Scale* ([15] p. 56) (Table 2) is a 12 item checklist for SS. The checklist was not initially designed to be a measure of the role of the SS member however, each item can be assessed using a Likert type scale and the assumption is that the higher the score rated by the participant the greater the agreement there is with the particular item. Further analysis may be required as this is the first time the Sessional Staff Role Scale has been used to assess understanding of the role of a sessional academic in teaching and learning.

**Table 2:** Sessional Staff Role items

	Item
1.	I identify my own professional development needs
2.	I actively engage in formal and/or informal professional development in learning and teaching
3.	I am familiar with, and keep up to date with, policies and procedures that affect my work
4.	I am aware of institutional student support such as academic skills programs, counselling, and disability services.
5.	I receive ongoing formal and informal feedback from the unit coordinator, peers and students
6.	I am aware of my roles and responsibilities as a sessional staff member
7.	I critically reflect (with myself and/or with others) on students’ learning, my teaching, and my professional development as a teacher
8.	I provide ongoing feedback to my department and unit coordinator
9.	I participate in, or contribute to, institutional/department/unit events and activities
10.	I am aware of opportunities in my school/university to gain recognition and reward for my contribution to quality teaching and learning.
11.	I am aware of departmental websites, learning management systems, discussion fora, and email
12.	I maintain regular and timely communication with my unit coordinator, department, and human resources.

### Ethical considerations

Approval for this study was obtained from the Western Sydney University Human Research Ethics Committee project number H11352 and the study was conducted according to ethical requirements. As data was collected using an online survey consent to participate was assumed by completion of survey. No names or identifying details were collected.

### Analysis

The psychometric details of each measure were examined using SPSS (IBM SPSS version 24 2016), for the participant’s response to job satisfaction, person-organisation fit (job fit), perceived organisational support (job support) and the sessional staff role scale (job role). Path analysis is an extension of multiple regression where the estimates of significance are demonstrable between the variables. This includes direction of effects and can provide some measure of causal modeling. The advantage of using Structural Equation Modelling (SEM) here is that it combines ‘path and factor analytic techniques in the one predictive model’ ([31] p. 2126). The use of the term model is as a descriptor of the relations amongst the variables as a statistical statement and the term path diagram is a pictorial representation of this model. Path analysis; where job satisfaction is treated as the endogenous (dependent) variable and job fit, job support, and job role are exogenous variables, is graphically created by the use of AMOS (IBM SPSS version 24 2016). Maximum likelihood estimation is utilised for normally distributed data such as ours as it is more ‘forgiving’ of a smaller sample [32].

## Results

### Psychometric testing of scales

The results begin with descriptive analyses of the measures used with this sample followed by path analysis and model restructure. Reliability assessment of the *Global Job Satisfaction Scale* [27] using Cronbach’s Alpha resulted in a score of 0.82 for the six items and all items were correlated at the 0.05 level (see Table 3). A Confirmatory Factor Analysis (CFA) of the total group (*N* = 133) utilising AMOS revealed a good model fit of > 0.90 (GFI of 0.97, RMSEA 0.07, chi-sq of 15.84 (df 9), and *p* = 0.07).

**Table 3:** Descriptive statistics and correlation estimates (N = 133)

Variable	Scale α	M(SD)	1	2	3	4
1. Global Job Satisfaction	0.82	4.50 (0.44)	–			
2. Perceived Organisational Support Scale	0.92	3.71 (0.71)	.403**	–		
3. Perceived-person organisation fit	0.89	3.76 (0.69)	.474**	.478**	–	
4. Sessional Staff Role Scale (revised)	0.82	4.01 (4.02)	.182*	.401**	.458**	–

Note: ^∗^ *p* < 0.05; ^∗∗^ *p* < 0.01

An initial Exploratory Factor Analysis (EFA) utilising Principal Axis factoring (PAF) was performed on the six item *Perceived Organisational Support Scale* by Eisenberger et al. (1986 *see* Table 3) as the item group were uniquely selected from the larger original scale of 36 items. This resulted in a one factor model with factor loadings ranging from 0.65 to 0.95, and which accounted for 67.49% of variance. A CFA revealed a good model fit (GFI of 0.97, RMSEA 0.05, chi-sq of 12.53 (df 9), and *p* = 0.16).

The three item *Perceived-Person Organisation Fit* by Cable and Judge 1996 *(see* Table 3) model is not identifiable using CFA as the degrees of freedom are 0 and the probability level cannot be computed [33]. An EFA was examined instead, again utilising PAF and this resulted in a one factor model with good factor loadings ranging from 0.75 to 0.94, and which accounted for a high 81.87% of variance.

The 12-item *Sessional Staff Role Scale* by Harvey and Fredericks 2015 *(see* Table 3) has no previous data to compare. This is a new measure arising from a ‘checklist’ created by Harvey and Fredericks in response to three areas – 1) assessing – achieving and sustaining good practice (items 1–7), 2) participating in the life of the institution/faculty (items 8–10), and 3) communicating with others regarding teaching (items 11 & 12). The authors were agreeable to the use of these items as a potential pilot test. A correlation matrix revealed all items were weakly to moderately related. The 12 items do not provide an adequate model fit (GFI < 0.81). However, as the authors had initially constructed their item list in three subscales. Items 1 to 7 (Achieving and sustain good practice) did provide a better model fit (GFI 0.92, RMSEA = 0.11 (Chi^2^ = 37.69, DF = 14, *p* = 0.001)).

Using items 8–12 did not provide an adequate model fit however, and item 12 was highly correlated with item 8 (> 0.8). as this was the first time the Sessional Staff Role Scale was used in a survey a decision was made to use the 11-item model for the measurement of Job Role. This includes the first 11 items in Table 2. A CFA with a one factor solution is gained from the 11 item model (VE = 49.85%).

#### Path analysis model

The goal of inference from this data was to determine if organisational support, organisational fit, and job role positively and significantly contributed to global job satisfaction. To this end a regression analysis was initially conducted and confirmatory models were created and interpreted. While the regression is a useful sequential method of estimating the relations amongst the variables, it does not take into account measurement error and covariance [32].

**Fig. 1:**
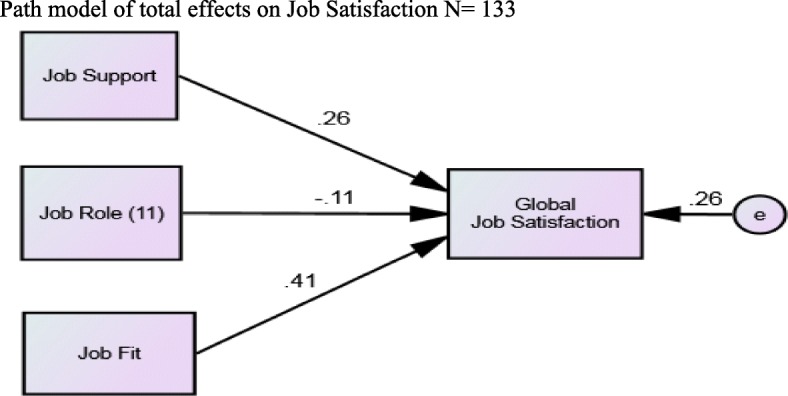
Chi-square (χ2) = 80.304. Degrees of freedom (df) = 3. Probability level (p) = .000. GFI = .77; CFI = .33; RMSEA = .44

The size of path coefficients (see Table 4) in the output path diagram demonstrate that Job Fit (as measured by PPOF) followed by Job Support (as measured by POSS) data have greater effects on Global Job Satisfaction. Parameter estimations for a path analysis are conducted using SEM. However, the model is such a poor fit to the data here the model is unacceptable using these parameters (Fig. 1).

**Table 4:** Regression Results using a Stepwise process (N = 133)

		Unstandardised Coefficients		Standardised Coefficients
Model		B	Std. Error	Beta
1				
	Job Support	.207	.065	.344^b^
	Job Fit	.458	.140	.404^a^
	Job Role11	−.025	.068	−.045

Note: dependent variable is Global Job Satisfaction

^a^significant at 1% level

^b^significant at 0.1% level

#### Nested model

**Fig. 2:**
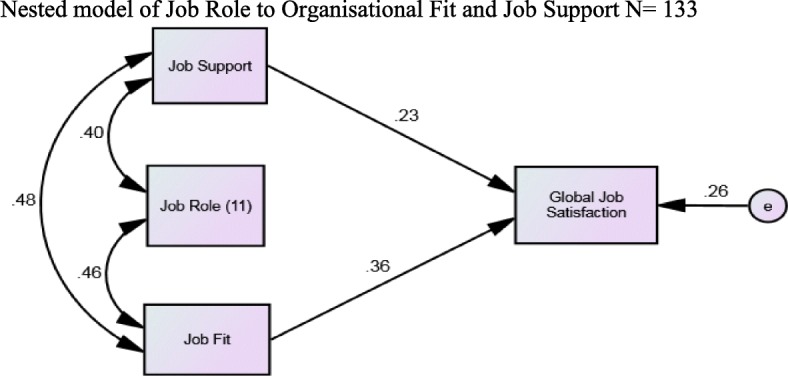
Chi-square = 1.433. Degrees of freedom = 1. Probability level = .231. GFI = .995; CFI = .988; RMSEA = .057

The three variables of Job Role, Job Fit and Job Support did not demonstrate an acceptable model despite statistically significant pathways. Therefore, an alternative model was tested and described (see Model 2 below). In this model, the Job Role variable is set to mediate through the Job Fit and Job Support variables. This is based on the premise that if a person is attempting to determine if they fit well with the organisation and have the forms of supports tailored to their work, then knowing what their role is would be a mediating factor rather than as an independent effect on Global Job Satisfaction as seen in the inadequate Model 1. Consequently, in this alternative model, Job Role should not be modelled as a main effect as it is in the first model. Job Role creates and supports Job Fit and Job Support in this hypothesis as a smaller model occurring within a larger model. This alternate approach creates a ‘nested’ model [34] and demonstrates good model fit indices and is thereby superior to the first model (Fig. 2).

## Discussion

In this study we hypothesised that organisational support, organisational fit and SS role will positively and significantly contribute to the job satisfaction of sessional academic staff. Through the use of SEM this has been demonstrated, albeit in an alternative model to that first proposed. Three of the four measures used in this study demonstrated good psychometric qualities with the fourth one (Job Role) requiring some adjustment.

### Global job satisfaction

The results of the sample revealed a statistically significant (0.02) mean score for satisfaction (4.50) when compared with the Gutierrez et al. score of 4.18 [28]. This was an important finding for us as recent literature on the current academic workplace portrays it as a stress ridden role with a somewhat gloomy outlook [35]. Baker [36] found that an increase in satisfaction was linked to retention of SS. However, job satisfaction is a dynamic and flexible construct [22] and as stated by Hagedorn ‘no single conceptual model can completely and accurately portray the construct’ ([23] p.6).

The global approach to a job satisfaction scale aimed to capture a ‘worker’s general affective reaction to the job without reference to any specific job facets’ ([37] p.50). There are many reliable and valid global job satisfaction measures but as Quinn and Sheppard pointed out nearly 50 years ago – many are occupation specific or ‘homogeneous’, or simply too long and complicated [37].

Our SS may or may not be registered nurses as a small number of the staff who teach science based subjects are science experts - not nurses. Use of an occupation specific tool such as the McCloskey Muller Satisfaction Scale (MMSS) [38] would be inappropriate in this study. There is also a perception that sessional teaching may be the beginning step needed for a full time academic career [39], and that boundaries of work and responsibilities may be blurred in an eager effort to appeal at job interviews. It is argued here however, that job satisfaction would be impacted by such plans and the results of this small study do not support this.

Staff selection is fraught with many contextual issues such as experiential and qualification issues. The contribution of Job Fit to Job Satisfaction (0.36 Fig. 2) demonstrates good selection processes and commitment to the job by the SS. In this study, more than half of the sample have an ongoing SS role each year- some in the first half of the calendar year, and others in the second half according to when their subjects are conducted. However, many new SS are assessed and employed each year. Job fit is important to job satisfaction. The question posed is - how much of job fit is achieved by the individual and how much is created through good employment selection and institutional processes?

Job support (POSS) demonstrated good psychometric properties and similar results in the path analysis to job fit. The question of whether a SS person feels supported in fulfilling their employment is essential to the management and organisation of SS. In this study, support is significantly related to job satisfaction. Needleman et al. [40] also found that support is critical to job satisfaction amongst tenured staff. This can be interpreted as ‘support increases satisfaction’. The implication of this finding is that, − in order to retain SS - an investment in support management is required. Institutional support however, is less well explored than educational delivery [40]. Mentorship and team collegiality are aspects of institutional structures that can provide support to the SS and thereby increase job satisfaction.

Research indicates that if support (be it perceived or actual) is not available to the new and even the seasoned academic staff person, the flow-on effect leads to lowered job satisfaction and lowered performance [40]. Baker also supports this stating that ‘high levels of empowerment and low levels of burnout were significant predictors of work satisfaction, with empowerment being the stronger predictor’ ([40] p. 413).

Recognition, inclusion, engagement and collegiality are some of the forms of supports needed for an inclusive workplace according to Rea [19]. The author claims that limitations of sessional work can cause financial as well as professional hardship. Rea states that ‘inequities and gross exploitation cannot be kept hidden as the dirty secret of the contemporary academic profession’ which lends a great deal of weight to the need for highly visible and structural support for SS ([19] p.13).

The staff role scale was the least successful tool used in this study. The 12 statements utilised performed poorly in CFA assessment, although the use of an 11 item model was found to work well within the model. Understanding the role of the SS is crucial to providing quality teaching and learning but the future may create entirely different blended and online learning environments. The role of SS will continue to change. Budget restrictions may target both ongoing and sessional nursing academics forcing some classroom education into on-line delivery thereby saving the organisation money or raise the costs of nursing studies [41].

#### Model

The results of modelling role, job fit and job support on job satisfaction were statistically significant for job fit and job support and non-significant for job role in the first model (see Fig. 1). The model did not demonstrate a good fit with the data producing unacceptable fit indices. What is not in this model may well be of greater importance to job satisfaction of SS. However, it is important for good SS management to explore the importance of support and job fit (right person right job) when numbers of casual staff now exceed those of permanent staff.

An alternate model examined the ‘mediation’ of Job Role through Job Fit and Job Support and demonstrated a good fit with the data as supported by high fit indices (see Fig. 2). Path analysis is used to refine the causal hypothesis. The path coefficient for job role is very small, so it made sense for us to eliminate the pathway. The new, ‘nested’ model, which has the same variables but fewer pathways, provided a more sensible outcome and good model fit. The strength of this model indicates a strong mediating effect of Job Role with Job fit and Job Support. This is based on the premise that if a person is attempting to determine if they fit well with the organisation then knowing what their role is likely to be would be is a strong intervening consideration.

#### Limitations

One of the strengths of this small study was the use of valid and reliable measures to explore potential effects on job satisfaction. However, results are limited by the sample size, sample type, and what is left out of the modelling such as career progression planning and workplace location, which may also have a significant impact on job satisfaction for SS. Correlations are between variables in this specific data set and cannot be generalised beyond this population.

Causality requires longitudinal data and as this was cross-sectional data, causal inference cannot be drawn. One issue of the small sample size is that all results should be read with caution if not rejected outright as SEM is particularly sensitive to sample size and those with *N* < 200 are ‘undesirable’ [31]. As Green states though – when variables are reliable, the model is simple, and the effects are strong, it may be acceptable if not rigorous practice to utilise a sample size such as ours. Green also points out Bentler and Chou’s [42] recommendation of applying at least 5 cases to each model parameter (5:1 ratio). As there are 10 model parameters our sample meets this criteria.

#### Future directions

This study indicates that job satisfaction for SS is predicated on having the right person for the job as well as adequate supports [43] for the academic role. In her study of casual teachers, Bamberry ([44] p. 49) claims ‘casual employment can erode the job quality of otherwise decent work within professional occupations’ yet this study does not demonstrate support for this claim. Further research could include use of these measures on full time staff as a group for comparison.

More than half of the curricula is delivered here by SS and according to Crimmins et al. [45] this trend is likely to increase in the future. Support for and selection of SS are important mediators of job satisfaction whereas SS role did not contribute to this small model with this sample. Job satisfaction relates to retention, which completes a round circle back to stability of staff. Replication of this study would be useful to address the current limitation of causality and incorporate Hierarchical Linear Modelling analyses for the issue of nested models, hierarchical structures, and for longitudinal data.

## Conclusion

An investigation into the relationships of support, job fit and job role on the global job satisfaction of SS staff has yielded important information that can be especially useful in recruiting and retaining valuable academic staff. Investments at all levels in universities must now be made to create a seamless team of full time, part time, and sessional academics or the system will fail our newest professionals. As the full-time academic disappears from our universities and sessional academics become the new normal, there are new imperatives in terms of maintaining high quality learning and learning outcomes. Knowing what the job role entails feeds into job fit and job supports, which are important in providing job satisfaction – an outcome that supports staffing stability, retention, and motivation.

## Additional file

Additional file 1: Sessional Staff Spring Satisfaction and Suggestion Survey. (DOCX 28 kb)

## Abbreviations

GJS: Global job satisfaction
M: Mean
POSS: Perceived organisational support scale
PPOF: Perceived-person organization fit
SD: Standard deviation
SEM: Structural equation modelling
SS: Sessional staff
