# Supplementary material for: Modelling job support, job fit, job role and job satisfaction for school of nursing sessional academic staff
Source: BMC Nurs. 2018 May 24;17:22. doi: 10.1186/s12912-018-0290-2 (PMC5968670; doi:10.1186/s12912-018-0290-2)
Supplement: Supplementary file 1 — Sessional Staff Spring Satisfaction and Suggestion Survey. (DOCX 28 kb) [file 12912_2018_290_MOESM1_ESM.docx]

**Sessional Staff Spring Satisfaction and Suggestion Survey**

Dear Sessional Staff member,

Thank you very much for spending time providing us with your thoughts and ideas on teaching and learning in the School of Nursing and Midwifery. We recognise that change and improvements come about by honest and open reflective opinions and we would love to have yours.

By now you will have read through the invitation to this study that outlines the project’s aims and methods. This survey is totally anonymous and information will remain confidential and used only for the purposes of improving our supports for you and thereby improving teaching and learning at the School of Nursing & Midwifery.

If you could please take a few minutes to tell us about the experience of working as a sessional teacher/marker.

If you would prefer to speak directly to the Director of Casual Workforce & Program Resource Allocation please phone Robyn Moroney on 02 4570 1931

Yours Sincerely

Leanne Cowin, Robyn Moroney and Kathleen Dixon

**Demographic and quality improvement questions (12 questions)**

Q1. What campus or campuses have you taught at this semester ?

- What year did you teach text box ____________________
- What units did you teach text box ____________________
- What course did you teach text box ____________________

Q2. Which units would you prefer to work in next year and why?

(text box) ____________________

Q3. How many years of clinical experience do you have?

(text box) ____________________

Q5. How long have you worked as a sessional staff member in the School of Nursing & Midwifery?

(text box) ____________________

Q6. Please broadly describe any problems or issues you experienced with Teaching/Marking this semester.

Description of issue/s (text box) ____________________

Not Applicable

Q7. Do you feel you were you adequately supported during the Autumn 2015 semester

Yes (text box) ____________________

No (text box) ____________________

Q8. Did you receive weekly course materials in a timely manner?

No

Yes, issues arising include: (text box) ____________________

Q9. Were the course (unit) materials detailed enough to know what was expected of you as a teacher?

Yes

No, issues arising include: (text box) ____________________

Q10. Were you able to understand and feel comfortable with the requirements for assessment – if not what would assist you with these requirements?

Yes

No (details)____________________

Q11. Did the marking time allocation match the time provided (in your contract) in your opinion? Why or Why not?

Yes (text box) ____________________

No (text box) ____________________

Q12. Are you interested in professional development? If you respond - yes, please write any specifics

No

Yes (text box) ____________________

**Succession building items (5 items text box for each)**

1. Would you take a permanent academic position in the School if offered and if no – why not
2. What are your short term career plans (3 years)
3. What are your long term career plans (10 Years)
4. How do you use the external feedback on your teaching and marking for professional development? (not applicable button included)
5. Would it be useful for you to have a mentor who can assist you to plan your academic career? (not applicable button included)

**Global Job Satisfaction Scale © Pond & Geyer 1991 (6 items)**

1. If you had to decide all over again whether to take the job you now have, what would you decide?

| Definitely not take job | Not take the job | Unsure | Take the job | Definitely take the job |
| --- | --- | --- | --- | --- |
| 1 | 2 | 3 | 4 | 5 |

(b) If a friend asked if he/she should apply for a job like yours with your employer, what would you recommend?

| Strongly not recommend | Not recommend | Neither | Recommend | Strongly Recommend |
| --- | --- | --- | --- | --- |
| 1 | 2 | 3 | 4 | 5 |

(c) How does this job compare to your ideal job ?

| Very far from ideal | Far from ideal | Unsure | Close to ideal | Very close to ideal |
| --- | --- | --- | --- | --- |
| 1 | 2 | 3 | 4 | 5 |

(d) How does your job measure up to the sort of job you wanted when you took it ?

| Not at all what I wanted | Not what I wanted | Unsure | What I wanted | Just what I wanted |
| --- | --- | --- | --- | --- |
| 1 | 2 | 3 | 4 | 5 |

(e) All things considered, how satisfied are you with your current job?

| Very dissatisfied | Dissatisfied | Unsure | Satisfied | Very satisfied |
| --- | --- | --- | --- | --- |
| 1 | 2 | 3 | 4 | 5 |

(f) In general, how much do you like your job?

| Not at all | Not at all sometimes | Unsure | A great deal sometimes | A great deal |
| --- | --- | --- | --- | --- |
| 1 | 2 | 3 | 4 | 5 |

**Perceived Organisational Support Scale © Eisenberger et al 1986 (6 items)**

| Strongly Disagree | Disagree | Neither Agree or Disagree | Agree | Strongly Agree |
| --- | --- | --- | --- | --- |
| 1 | 2 | 3 | 4 | 5 |

1. The School of Nursing & Midwifery takes pride in my accomplishments
2. The School of Nursing & Midwifery really cares about my well-being
3. The School of Nursing & Midwifery values my contributions to its well-being
4. The School of Nursing & Midwifery strongly considers my goals and values
5. The School of Nursing & Midwifery shows little concern for me.
6. The School of Nursing & Midwifery is willing to help me if I need a special favour.

**PPOF, perceived-person organization ﬁt © Cable et al 1996 (3 items)**

| Not at all | A little | Neutral | A lot | Completely |
| --- | --- | --- | --- | --- |
| 1 | 2 | 3 | 4 | 5 |

1. To what degree do you feel your values ‘match’ or fit this organization and the current employees in this organisation?
2. My values match those of current employees in organisation
3. Do you think the values and ‘personality’ of this organisation reflect your own values and personality?

**Sessional Staff Role Scale © Harvey & Fredericks (2015) p. 56 (12 items)**

| Strongly Disagree | Disagree | Neither Agree or Disagree | Agree | Strongly Agree |
| --- | --- | --- | --- | --- |
| 1 | 2 | 3 | 4 | 5 |

1. I identify my own professional development needs
2. I actively engage in formal and/or informal professional development in learning and teaching
3. I am familiar with, and keep up to date with, policies and procedures that affect my work
4. I am aware of institutional student support such as academic skills programs, counselling, and disability services.
5. I receive ongoing formal and informal feedback from the unit coordinator, peers and students
6. I am aware of my roles and responsibilities as a sessional staff member
7. I critically reflect (with myself and/or with others) on students’ learning, my teaching, and my professional development as a teacher
8. I provide ongoing feedback to my department and unit coordinator
9. I participate in, or contribute to, institutional/department/unit events and activities
10. I am aware of opportunities in my school/university to gain recognition and reward for my contribution to quality teaching and learning.
11. I am aware of departmental websites, learning management systems, discussion fora, and email
12. I maintain regular and timely communication with my unit coordinator, department and human resources.

**The end**

**Thank you very much for your time and effort.**
